# LPS-Induced Genes in Intestinal Tissue of the Sea Cucumber *Holothuria glaberrima*

**DOI:** 10.1371/journal.pone.0006178

**Published:** 2009-07-08

**Authors:** Francisco Ramírez-Gómez, Pablo A. Ortiz-Pineda, Gabriela Rivera-Cardona, José E. García-Arrarás

**Affiliations:** Department of Biology, University of Puerto Rico, Río Piedras, San Juan, Puerto Rico; National Institute on Aging, United States of America

## Abstract

Metazoan immunity is mainly associated with specialized cells that are directly involved with the immune response. Nevertheless, both in vertebrates and invertebrates other organs might respond to immune activation and participate either directly or indirectly in the ongoing immune process. However, most of what is known about invertebrate immunity has been restricted to immune effector cells and little information is available on the immune responses of other tissues or organs. We now focus on the immune reactions of the intestinal tissue of an echinoderm. Our study employs a non-conventional model, the echinoderm *Holothuria glaberrima*, to identify intestinal molecules expressed after an immune challenge presented by an intra-coelomic injection of lipopolysaccharides (LPS). The expression profiles of intestinal genes expressed differentially between LPS-injected animals and control sea water-injected animals were determined using a custom-made Agilent microarray with 7209 sea cucumber intestinal ESTs. Fifty (50) unique sequences were found to be differentially expressed in the intestine of LPS-treated sea cucumbers. Seven (7) of these sequences represented homologues of known proteins, while the remaining (43) had no significant similarity with any protein, EST or RNA database. The known sequences corresponded to cytoskeletal proteins (Actin and alpha-actinin), metabolic enzymes (GAPDH, Ahcy and Gnmt), metal ion transport/metabolism (major yolk protein) and defense/recognition (fibrinogen-like protein). The expression pattern of 11 genes was validated using semi-quantitative RT-PCR. Nine of these corroborated the microarray results and the remaining two showed a similar trend but without statistical significance. Our results show some of the molecular events by which the holothurian intestine responds to an immune challenge and provide important information to the study of the evolution of the immune response.

## Introduction

In metazoans, the immune system is usually associated with specialized cells that are found distributed in the vascular system and within most tissues. Human lymphocytes and invertebrate hemocytes/coelomocytes are examples of circulating cells specialized in immune defense. However, certain organs are also known to participate in immune responses. For example, the human liver is associated with innate responses and its role as an immune organ is being increasingly investigated [1], [2]. In teleost fishes, the kidney is an important immune organ where B-lymphocytes originate and differentiate [3] and in humans it is the subject of numerous studies on autoimmune disease and transplantation immunology [4], [5]. Even organs not commonly associated with immunity can respond to an immune challenge and participate in the immune response. For example, the central nervous system is clearly affected by the immune state of the organism, playing a role in controlling body temperature and other immuno-modulatory responses [6], [7]. Likewise, in invertebrates, immune cells can be produced in definite lymphoid-like organs, i.e. the fat body of insects [8], but in some groups it has been shown that the immune function is shared or distributed by organs and tissues in different areas of the body (internal epithelia, gastric and excretory tissues) [9]. The digestive tract comprises one of these organs that play an active role in the immune response as a barrier against pathogens that might be present or even harbored within its luminal cavity [10]. Furthermore, mucosal immunity is even more active than systemic immunity due to the overwhelming antigenic load that enters daily in the form of food antigens and commensal bacteria [11]. Additionally, circulating immune cells are able to infiltrate the gut tissues participating in intestinal immune reactions, as occurs with vertebrate lymphocytes and very likely with invertebrate coelomocytes.

Studies of immune systems in non-vertebrates have been limited to certain metazoan groups, being the class Echinoidea of the phylum Echinodermata one of the most comprehensively studied. Being deuterostomes, echinoderms comprise the sister group of chordates. Thus, studies of the echinoderm immune responses are important in determining the evolution of the immune system in metazoans. In echinoderms the main immune effector cells are the coelomocytes. While no definite lymphoid organ has been found, the source of coelomocytes has been pointed towards the axial organ, the haemal system, the polian vesicles, the dermal connective tissue or the coelomic epithelia [12], [13]. In recent years, the molecular basis of echinoderm immune systems has been greatly advanced. In particular, two types of data have been generated of importance to echinoderm immunity. First, is the publication of the sea urchin genome that has provided insight into the echinoid immune repertoire. Some of the findings have changed our paradigms about comparative immunity [14], [15]. Second, is the identification of genes expressed in sea urchin coelomocytes following immune challenge with bacterial lipopolysaccharides (LPS). Some examples of immune system associated molecules include: complement molecules, lectins, serine proteinase inhibitors, scavenger receptors (SRCRs), cytoskeletal proteins, clotting molecules and the highly variable family of effector molecules 185/333 [16], [17], [18], [19], [20], [21]. However, studies of the echinoderm immune system have been mainly limited to the study of only one of the echinoderm classes, the Echinoidea, and mostly to the expression of immune genes by one cell type, the coelomocytes.

The present study uses another echinoderm species, the sea cucumber *Holothuria glaberrima*, to identify molecules expressed in the intestine upon immune activation. In contrast to the sea urchin (Echinoidea), sea cucumbers are members of the class Holothuroidea, which diverged from the echinoids between 500–600 million years ago [22], thus providing for comparative analyses with the sea urchin and for the identification of new molecules associated with echinoderm immune responses. Our work is based on a database of over 7000 expressed sequenced tags (ESTs) isolated from three cDNA libraries of intestinal tissues. In previous publication we reported the presence of several immune-related genes in the EST database: a serum amyloid A (SAA) protein [23], three serine proteinase inhibitors, a C-type lectin, a transglutaminase, several fibrinogen-like proteins, a ferritin, and proteins of the transferrin superfamily, to name a few. We also showed that these molecules are expressed by coelomocytes and that some of them are induced following an LPS challenge, thus confirming their role as immune molecules [24].

We have now used microarray technology to study the expression of these ESTs by establishing a profile of genes expressed in the intestine following an LPS challenge. Our results show that 50 genes are differentially expressed between the intestines of naïve and LPS challenged animals. Some of these belong to diverse functional categories: metal ion metabolism/transport, cellular metabolism, cytoskeleton function, and defense/recognition.

When compared to our previous study in coelomocytes [24], the differences in gene expression profiles are evident, reflecting the way each tissue (intestine vs. coelomocytes) respond towards an immune challenge. These data not only provide important information on the role of the intestine in immune responses but also increase our knowledge on the molecular evolution of the immune system.

## Materials and Methods

### Animals and treatments

Adult sea cucumbers (10–12 cm long) were collected from the northeastern rocky shores of Puerto Rico. Animals were kept in seawater aquaria at 20–24°C, for one week prior to the studies. Eighteen animals were used (9 per treatment and control) to obtain the mRNA necessary for array hybridization and PCR validation. Control animals were injected with 0.5 mL of filtered seawater. Treatment animals were injected once with 1 mg LPS diluted in 0.5 mL filtered seawater, as reported previously [24]. Once injected, animals were kept in the aquaria for 48 hours. The day of dissection, sea cucumbers were anesthetized in cold seawater (4°C) for 45 min, and their intestines removed for RNA processing. Briefly, a lateral incision was made between the division of the ambulacral feet and the dorsal part of the body, then, a portion (1 cm approx) of the large intestine was dissected for RNA extraction.

### RNA extraction

Dissected intestines were placed in RNAlater® (Applied Biosystems/Ambion, Austin, TX) solution and stored at 4°C for at least 24 h. RNA was extracted using a combination of the Chomczynski (1993) method using Tri-reagent® (No. 93289, Sigma, St Louis, MO) and the RNAeasy mini kit from Qiagen (Valencia, CA). Briefly, after homogenization with Tri-reagent and phase separation, the upper aqueous phase was mixed with 70% ethanol and added to the RNAeasy column. Then the RNA was purified according to the manufacturer's instructions. RNA concentration and purity was determined using a Nanodrop™ spectrophotometer (Thermo Fisher, Waltham, MA). Within the treatment and control groups, RNA for every 3 animals resulted in 3 pooled RNA samples per treatment group.

### RNA amplification and labeling

Agilent's low RNA input linear amplification kit PLUS was used to generate fluorescent cRNA (complementary RNA) for the microarray hybridizations, following the manufacturer's instructions. A total of 300 ng of labeled cRNA per sample was used for the hybridization.

### Microarray fabrication

A total of 7209 ESTs from three intestinal cDNA libraries were chosen for the microarray construction. The cDNA libraries were made from normal and regenerating (3 and 7-days post evisceration) intestines (details can be found in a previous publication [25]). Briefly, the evisceration process is induced in the sea cucumber by injecting 3–5 mL of KCl (0.35 M) into the coelomic cavity, after which the animal expels its internal organs. After evisceration, the animal regenerates its intestine, having an apparently functional organ after approximately four weeks (reviewed in [26]). Arrays were custom made by Agilent (Santa Clara, CA) using the eArray design tool and the SurePrint technology for printing. A design of 60-mer probes in a 15 k format was chosen, with 8 arrays per slide. Two or three 60-mer probes were designed per EST, and all probes were printed on the array to account for technical replicates. In addition to the sea cucumber ESTs, other echinoderm sequences available in the databases were also printed on the array: 75 ESTs from *Apostichopus japonicus*, 4 from *Parastichopus parvimensis* (both species are sea cucumbers) and 329 sequences from the genome of the sea urchin *Strongylocentrotus purpuratus*. Other non-echinoderm sequences were also printed, which included: mouse (3 sequences), rat (2), human (4), axolotl (5) and zebrafish (6).

### Microarray hybridization

Hybridizations were carried out at 65°C for 17 hrs in a rotating oven (Agilent, Santa Clara, CA). Post-hybridization washes were conducted according to Agilent's two-color microarray-based gene expression analysis protocol (Version 5.5, February 2007). Slides were scanned with an Agilent microarray scanner and data was obtained through Agilent's feature extraction software (Version 9.5.3.1).

### Array data analysis

The data was analyzed in R software with the Limma package from Bioconductor (http://www.R-project.org, www.bioconductor.org). For normalization purposes [27] MA-plots were generated representing the (R,G) data (R = red for Cy5 and G = green for Cy3), in which the log ratio of R versus G (M value = log_2_ R/G) was plotted against the overall intensity of each spot (A value = log_2_√(R×G). Within-array normalization was first applied and M-values were normalized within each array using the Global Loess Normalization method. Aquantile normalization was then applied to the A-values as a method for between-array normalization, to assure that the intensities and log-ratios had similar distributions across arrays. To estimate the average M-value for each gene and assess differential gene expression, a simple linear model was fitted to the data, and M-value averages and standard deviations for each gene were obtained. To find genes with significant expression changes between treatments, empirical Bayesian statistics were applied to the data by moderating the standard errors of the estimated M-values. P-values were obtained from the moderated t-statistic and statistical significance was set at p<0.05.

### Sequence analyses

Each EST was queried against the non-redundant protein database at the National Center for Biotechnology Information (NCBI, Bethesda, MD) using the BLASTX and BLASTP algorithms [28]. In both cases, the default BLAST parameters were used. Domains were searched with RPS-BLAST against the conserved domain database (CDD) [29] from NCBI. Alignments were performed using ClustalW [30] and edited with GeneDoc (v2.6.003) [31].

### Reverse transcription

The Improm-II reverse transcription system (Promega, Madison, WI) was used to synthesize cDNA, with 1 ug of total intestinal RNA and an oligo (dT)_15_ primer, according to the manufacturer's instructions. Appropriate RT negative controls were included (without reverse transcriptase) to determine the presence of genomic DNA contamination. Samples with genomic contamination were treated with TURBO DNA-free kit (Applied Biosystems/Ambion, Austin, TX) following manufacturer's instructions.

### Microarray validations

Primers for PCR validations were designed using Primer-3 software [32] and checked for hairpins and dimers using Netprimer software (PREMIER Biosoft, Palo Alto, CA), and synthesis was done by Alpha DNA (Montreal, Quebec). Primer sequences are presented in Table S1. Primers were designed to possess a Tm around 58°C to allow amplification with the same cycling program. The number of cycles for amplification was determined empirically to allow quantification in the linear range of PCR. After reverse transcription, 1/10^th^ of the cDNA was used for each PCR reaction with 0.2 uM of each primer, 100 uM dNTPs, 2 mM MgCl_2_, and 1 U Taq polymerase (Promega, Madison, WI). Cycling conditions were the same for all primer pairs: 94°C for 2 min, and then 30 cycles at 94°C for 30 s followed by 55°C for 45 s and 72°C for 45 s. PCR was carried out in MJ Research thermocyclers (now Bio-Rad) (either PTC-100 or PTC-200). PCR products were electrophoresed in 1% agarose gels stained with ethidium bromide and documented using Bio-Rad's GelDoc system (Hercules, CA). Densitometric analysis was done using Bio-Rad's Quantity One software. Statistical significance was tested using Student's *t* test.

## Results

### Differentially expressed genes in LPS challenged animals-

Gene expression analyzes were performed using 3 arrays, representing 3 biological replicates with one replicate done in a dye-swap manner to test for dye-based bias. The technical performance of the arrays was tested using three approaches: First, by analyzing the behavior of the array internal controls; second, by determining if any bias for either dye (Cy3 or Cy5) was present; and third, by the performance of the non-*H. glaberrima* sequences printed on the array.

For the first technical test, Agilent's microarrays contain a series of internal controls (SpikeIns) that allow monitoring the technical performance of the array, in terms of linearity, sensitivity and accuracy. The 536 internal controls behaved as expected, e.g., SpikeIn E1A_r60_n9 that should have appeared bright green on the array and should have had the lowest expression level, effectively did so (Figure S1A and C). Agilent's feature extraction software also performs a linear regression of the SpikeIns expected values versus the observed values to show the complete behavior of the controls. The data from this regression adjust to a linear model with a coefficient (R^2^) of 9.85 (Figure S1B).

For the second performance test, an MA plot was the best representation for visualizing the effect of global dye-bias normalization [33] and in our case this effect was almost null. The MA plot distributions of raw and normalized data were almost identical (Figure S2), suggesting that there was no significant technical variation. Similar labeling patterns were observed between the dye-swaps, showing again no dye-based bias (Figure S3). The dye-swap replica was made with a different set of animals, with the aim of using it not only as a technical replica but also as a biological replica.

The printing of non-holothurian sequences on the array also helped to confirm the specificity of the assay. From a total of 428 sequences from diverse organisms none of them showed significant expression levels. Among these sequences were several immune genes from the sea urchin genome, i.e., several TLRs, complement genes, 185/333 genes, RAG-like genes, NLR genes, NFkB and PGRPs; and one fibrinogen-like protein from the sea cucumber *P. parvimensis*. None of the sea urchin genes had a homologue among the printed EST sequences of *H. glaberrima*. The only sequence with a holothurian homologue was the *P. parvimensis* Fib-like protein. This sequence was approximately 46% identity to the FreD-containing proteins of *H. glaberrima*. However, the 2 probes designed for the *P. parvimensis* sequence did not match any region of the H. glaberrima sequences. The lack of significant signals for the non-*H. glaberrima* probes indicated the specificity of the array even for related sequences. In summary, these three tests indicated the high quality technical performance of the microarrays, resulting in reliable data for further analyses.

**Figure 1:**
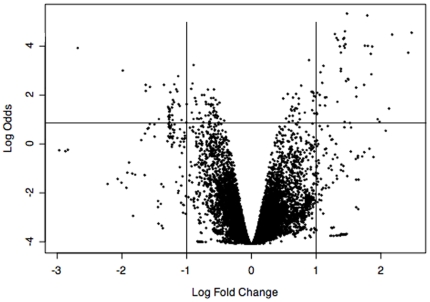
Volcano plot of statistical significance (Log Odds) versus fold change in intensity. Vertical lines indicate the two-fold change threshold, up (>1) or downregulation (<−1), to determine differentially expressed genes. Points below the horizontal line are not statistically significant. Each dot represents a single probe on the microarray. Dots located in the upper-left and upper-right cuadrants represent differentially expressed probes (down-regulated and up-regulated, respectively) at P<0.01.

The statistical analysis showed that there were few differences between the intestines of LPS-injected and control animals, as seen in the volcano plot (Figure 1). The vast majority of points were distributed in the central zone of the graph, where the Log fold change was less (between −1 and 1). Few points fell outside this zone (log changes >1 or <−1) and approximately half of these points showed high statistical significance (Log Odds >1). These points, located in the upper-right and upper-left quadrants of the volcano plot, represented a total of 136 probes, which showed significant differences in intensities (P>0.01) between intestines of LPS-treated animals and controls. According to the plot, the up-regulated probes (Log fold changes >1) showed a broader distribution with higher Log odds, in contrast to the downregulated (log fold change <−1), which were more compact toward the center. This was evident when the differentially displayed probes were further analyzed. The up-regulated probes showed P-values that were as low as 0.005, while none of the down-regulated probes showed P-values of less than 0.01 (Table S2).

The design of the microarray included 2 probes (sometimes 3) per each EST, therefore each EST was represented twice or three times on the slide. To determine which ESTs were differentially expressed, this redundancy was taken into account. Matching of the 136 differentially displayed features with their corresponding ESTs resulted in a total of 67 ESTs. Thirty (30) of these ESTs were assembled into 13 different contigs, while the remaining (37) corresponded to singlet unique sequences. In total, the 136 differentially displayed features on the array corresponded to 50 different sequences. Ten (10) additional probes were also differentially displayed on the array. These probes matched to 5 different contigs, but they were rejected from analysis due to their low representation of differentially expressed probes. These 5 contigs were “true contigs” in terms of their sequence, showing a uniform distribution of the ESTs that formed them without gaps in their sequences. However, for these contigs less than 10% of their probes showed differential expression and sometimes, similar probes even showed different expression levels. Thus they were excluded from the group of genes differentially expressed in the intestine following LPS injection.

From the 50 chosen sequences, 21 were up-regulated (fold change ≥2.0) in LPS treated animals and 29 were down-regulated (fold change ≤−2.0) (Table 1). Seven (7) of these 50 ESTs matched with known genes found in the databases, these are shown on Table 1: Major yolk protein (Myp), glycine-N-methyltransferase (Gnmt), fibrinogen-related protein (FREP), S-adenosyl-L-homocysteinase (Ahcy), alpha-actinin-2 (Actn-2), GAPDH and an isoform of Actin previously described by our group (Hg_Act1). According to their reported function these genes were categorized into: metabolic genes (GAPDH, Ahcy and Gnmt), cytoskeletal genes (Hg_Act1, Acnt2), metal ion transport/metabolism (Myp) and recognition/defense (FREP). Upon LPS treatment, four of these were up-regulated (Myp, Gnmt, FREP, Ahcy), while the remaining three (Actn, GAPDH and Hg_Act1) were underexpressed. The 43 remaining ESTs showed no homology to sequences on the databases including protein, ESTs and RNAs databases. However, 14 of these had a recognizable ORF (>40 amino acids) (Table 1).

**Table 1:** Differentially expressed sequences in the intestine after an LPS challenge. Acsn #: NCBI's accession number of the corresponding EST.

Up-regulated (fold change ≥2.0)					Down-regulated (fold change ≤−2.0)					
**ESTs with known identity**										
**P value**	**EST ID**	**Acsn #**	**Homology**	**e-value**	**P value**	**EST ID**	**Acsn #**	**Homology**	**e-value**	
0.00684	C5087-1		Major yolk protein	1e-49	0.04805	P3DP22F02	ES725371	A-Actinin	1e-83	
0.0309	P7DP04E06	ES726637	Glycine-N-methyltransferase	5e-29	0.0432	P7DP32B09	ES727907	GAPDH	3e-54	
0.03093	PNLP17D06	ES729344	FREP	2e-27	0.0451	C4705-2	FJ455438	Hg_Actin1		
0.03773	P7DP02C08	ES726499	S-Adenosyl-L-homocisteinase	2e-96						
**ESTs with no known identity (ORF>40aa)**										
**P value**	**EST ID**	**Acsn #**	**ORF size**		**P value**	**EST ID**	**Acsn #**	**ORF size**		
0.00684	C4874-1		123		0.02343	C539 -1		115		
0.00684	C4582-1		120		0.02343	C5501 -1		42		
0.00684	C5242-1		274		0.02668	P7DP42F11	ES728287	144		
0.01062	C927-1		40		0.02669	P7DP08F07	ES726827	145		
0.0124	C5028-1		43		0.02669	PNLP10F03	ES728876	110		
0.02932	PNLP14B02	ES729080	75		0.02932	P3DP18D04	ES725147	112		
0.0456	PNLP07A02	ES728683	62		0.03773	C4765 -1		58		
					0.03786	P7DP25G11	ES727585	43		
					0.04905	PNLP17B07	ES729325	101		
**ESTs with no recognizable ORF**										
**P value**	**EST ID**	**Acsn #**	**PolyA**		**P value**	**EST ID**	**Acsn #**	**PolyA**		
0.00552	PNLP09D04	ES728794	yes		0.01613	PNLP17C12	ES729338	no		
0.00684	P7AP03G08	ES725972	no		0.01613	P7AP07B02	ES726177	no		
0.00762	P3DP19C11	ES725198	yes		0.02668	P3DP09F12	ES724855	no		
0.01062	PNLP13E06	ES729033	yes		0.02669	PNLP26B02	ES729936	no		
0.03093	C4626-1		no		0.03335	P7DP20E01	ES727280	no		
0.03335	P7AP01A08	ES725801	no		0.03773	P3DP14F03	ES724974	yes		
0.03687	PNLP12E07	ES728982	no		0.03781	P7DP32C11	ES727919	no		
0.0451	P7AP02B05	ES725870	no		0.03899	P7DP01C01	ES726436	no		
0.0451	C2141- 2		no		0.03899	P3DP15A05	ES724982	yes		
0.04963	PNLP26A01	ES729925	no		0.04278	C2794 -1		no		
					0.04564	P7AP07G08	ES726209	no		
					0.04564	P7AP09A02	ES726274	no		
					0.04564	P7DP30C11	ES727789	yes		
					0.04564	PNLP11E04	ES728925	no		
					0.04658	P3DP15F10	ES725016	yes		
					0.04805	P7DP29A04	ES727691	yes		
					0.04963	P7DP10D05	ES726915	no		

### Sequence analysis

To determine the identity of the analyzed ESTs, their sequences were compared to those in the available databases (protein, ESTs and RNAs). ClustalW alignments were performed in certain cases to determine homologies and conserved domains/residues. The ESTs with known homologies included C5087-1 (Myp), P7DP02C08 (Ahcy), P7DP04E06 (Gnmt), P7DP32B09 (GAPDH), P3DP22F02 (Actinin), C4705-2 (Hg_Actin-1) and PNLP17D06 (FREP).

Contig 5087-1 (Myp) showed similarity to a major yolk protein from sea urchin as reported previously by our group [24]. The contig was 914 bp long and composed of 3 ESTs. It represented the last 237 amino acids (carboxyl end) of the protein and also contained the 3′ UTR. The echinoderm MYP is a large protein of approximately 1350 amino acids long. The 237 amino acids of the holothurian shared 44% identity with MYPs from other echinoderms. A phylogenetic tree made with several proteins of the transferrin superfamily clustered the sea cucumber MYP with the other echinoderm MYPs, showing that this sequence represents a homologue of MYP [24].

**Figure 2:**
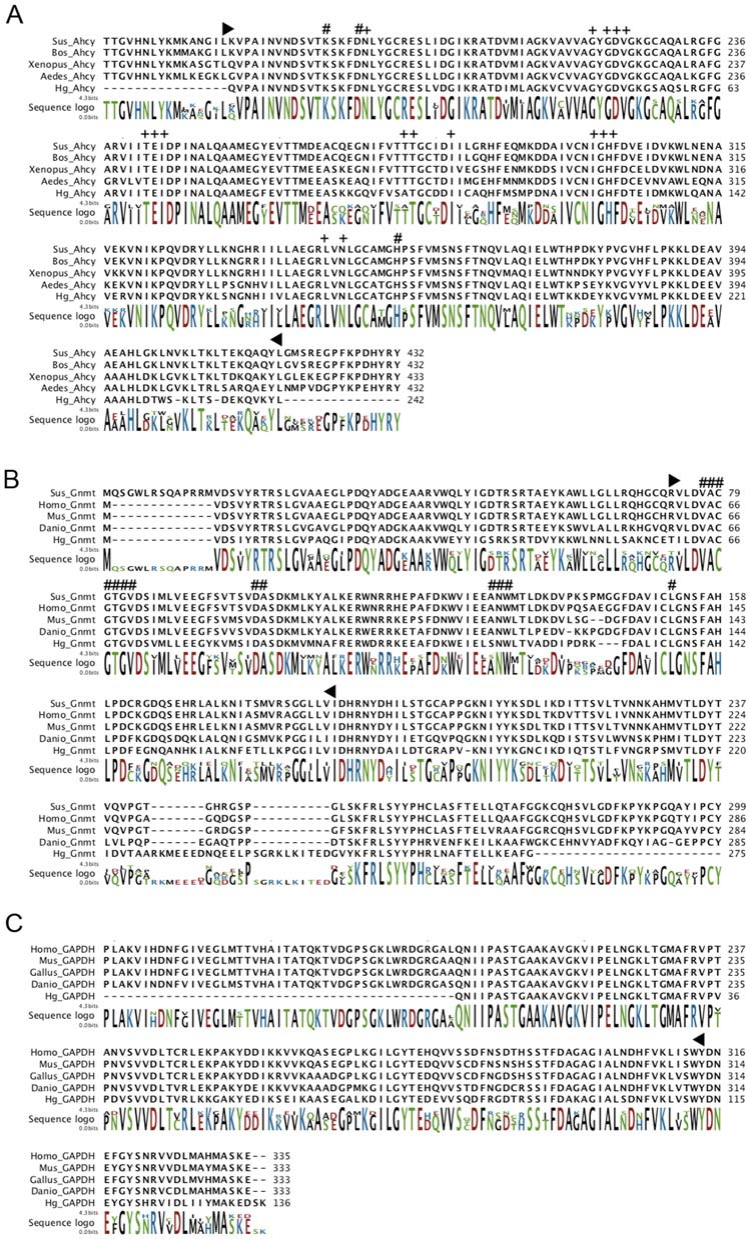
Sequence alignment of the metabolic genes found in the array. Weblogo is also shown to denote residue conservation levels. A. Alignment of Ahcys from *H. glaberrima*, *Bos Taurus* (77735583), *Aedes aegypti* (157118541) *Sus scrofa* (AJ427478.2) and *Xenopus tropicalis* (113205576). Numerals represent the active site of the enzyme; plus signs represent NAD+ binding sites. The AdoHcyase domain (cl09931) is enclosed between black arrowheads. B. Alignment of Gnmts from *H. glaberrima*, *Homo sapiens* (NM_018960.4), *Mus musculus* (15679953), *Sus scrofa* (NM_001110419.1) and *Danio rerio* (NM_212816.1). Numerals marks AdoMet binding sites. Black arrowheads enclose the AdoMet_MTases superfamily domain (cd02440). C. Alignment of GAPDHs from *H. glaberrima*, *Homo sapiens* (NM_002046.3), *Gallus gallus* (NM_204305.1), *Mus musculus* (NM_008084.2), and *Danio rerio* (NM_001115114.1). The black arrowhead represents the ending of the C-terminal domain of GAPDH (pfam02800).

Three sequences (Ahcy, Gnmt and GAPDH) were found with high similarities to metabolic enzymes. The ESTs representing these enzymes showed high levels of amino acid identity, as well as the characteristic domains of these proteins. They also showed a high degree of conservation when compared to sequences in several other organisms. The EST similar to Ahcy (P7DP02C08) encoded the last 225 amino acids (carboxi terminal) of the protein (as well as the 3′UTR), which is 432 amino acids long according to the databases. The sequence showed a domain belonging to the AdoHCyase superfamily (cl09931) (e-value 9e-103) and also showed several binding sites for NAD+ and oligomerization interfaces. The sea cucumber Ahcy has been well conserved as evidenced by a multiple sequence alignment with other Ahcys from cow, frog and mosquito (Figure 2A). The EST for Gnmt (P7DP04E06) encoded the full sequence for the protein (295 amino acids), including 5′ and 3′UTRs. This sequence showed a high conservation level of 65% identity when compared to other Gnmts from mouse, human, fish and pig (Figure 2B). It also had a domain belonging to the AdoMet_MTases superfamily of proteins (cl09112) and several binding sites for S-adenosylmethionine. Finally, the third metabolic gene corresponded to GAPDH, which was represented by EST P7DP32B09. This EST encoded 136 amino acids of the C-terminal of the protein and the 3′UTR for the mRNA. The average size of GAPDH in other organisms is 333 amino acids, so this sequence represented 40% of the complete protein. When comparing this fragment to other GAPDHs from various organisms (human, mouse, chicken and fish) it showed 80% amino acid identity. Additionally, it harbors the C-terminal domain of the protein (pfam02800) (Figure 2C).

**Figure 3:**
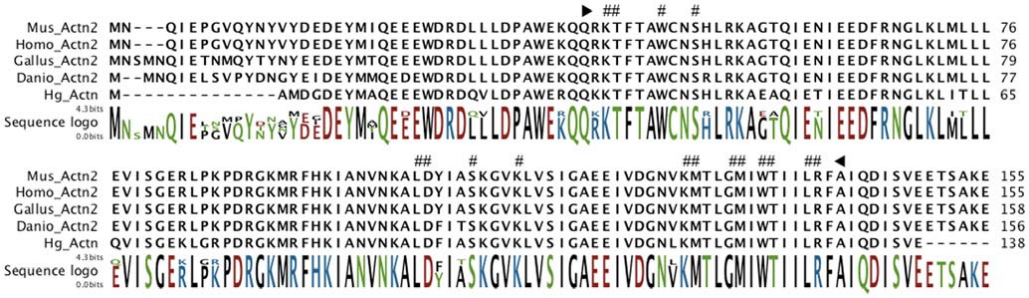
Multiple sequence alignment of Aplha Actinins from *H. glaberrima*, *Homo sapiens* (CH471098.1), *Mus musculus* (AY036877.1), *Gallus gallus* (NM_205323.1) and *Danio rerio* (NM_001037573.1). Enclosed in black arrowheads is the first calponin homology domain (cd00014) of the protein. Numerals denote putative actin binding sites.

Two sequences (C4705-2 and P3DP22F02) were found to represent cytoskeletal proteins. The first sequence, contig C4705-2, represented an actin isoform previously described by our group as Actin 1 (accession FJ455438). The second EST (P3DP22F02) encoded for 140 amino acids similar to an alpha-actinin. These amino acids were located at the N-terminal of the protein, having the initial Met and also the 5′UTR. When aligned with other alpha-actinins from diverse organisms (mouse, human, chick and fish) there is approximately 88% identity showing a high level of conservation. The sequence also contained the first calponin homology (CH) domain (cd00014) and several actin-binding sites typical of these proteins (Figure 3). Alpha-actinin is approximately 890 amino acids long in other species; hence the holothurian fragment represented 16% of the total sequence. Unfortunately, we did not obtained the complete sequence, because the insert size of clone P3DP22F02 was only 578 base pairs. This represented one of many incomplete clones that were present in the library of 3 days-post evisceration due to an effect of library construction.

The EST PNLP17D06 encoded a protein with a fribrinogen-related domain (FreD). The highest homology search resulted in a fibrinogen-like precursor (FREP-A, accession P19477) from another sea cucumber (*Parastichopus parvimensis*) with an e-value of 3e-27 and 46% amino acid identity. The holothurian FREP (PNLP17D06) corresponded to 270 amino acids at the C-terminus of the protein. This sequence lacked the initial Met and the corresponding 5′ UTR, but it did include the stop codon and 3′UTR. This sequence was reported previously where it was compared to other fibrinogen-related proteins from various organisms [24]. There we showed that the fibrinogen domain of the C-terminal portion was well conserved as well as key cysteine residues for disulfide bonds.

### Validation data for selected genes by semi-quantitative relative RT-PCR

**Figure 4:**
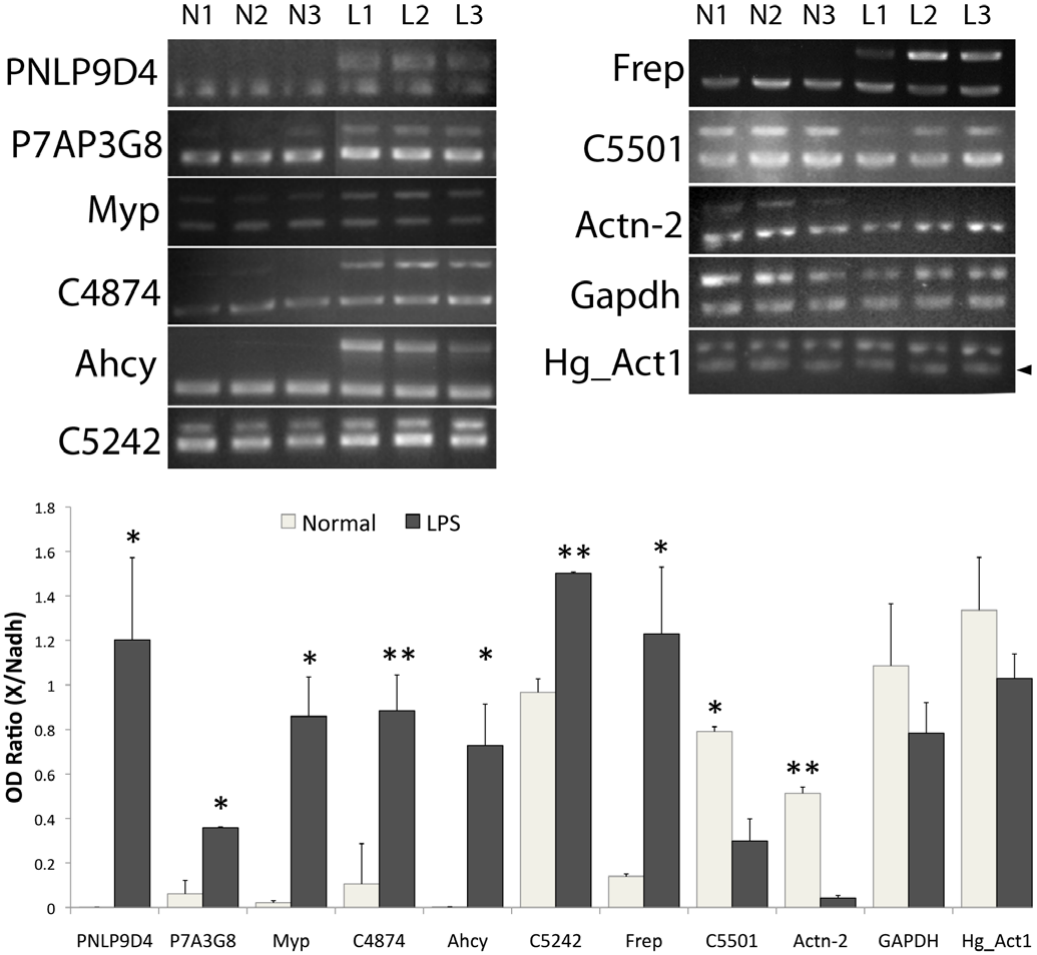
Semiquantitative relative RT-PCR validation of 11 selected sequences. For each sequence a gel image shows the PCR amplification of the gene and the control NADH (second lower band), except for Hg_Act1, where the actin band is lower than that of NADH. Each lane represents RT-PCR products from RNA pooled from three different animals. Graph bars indicate the averaged OD ratios between each gene and NADH for three different experiments (each with a different pool of animals). Lines represent standard deviation. Asterisks represent t-test significance (*P<0.05, **P<0.01).

To validate array results, 11 ESTs were chosen for RT-PCR analysis and their expression levels were measured relative to a housekeeping gene. These ESTs included 6 ESTs with known homology (Myp, Ahcy, Frep, Actn, Gapdh, Hg_Act1) and 5 ESTs with no known homology (PNLP09D04, P7AP3G8, C4874, C5501 and C5242). The housekeeping gene NADH dehydrogenase was used as control for normalization. This gene remained unchanged in the array (P = 0.998) and previous work has demonstrated that its expression levels remained constant in holothurian intestines during regeneration [25]. Gel images and quantitation graphs are presented in Figure 4. According to the array, the genes Myp, Ahcy, Frep, PNLP09D04, P7AP3G8, C4874 and C5242 were up-regulated upon LPS treatment (Table 1), and according to the RT-PCR analysis, all of them were consistently up-regulated when compared to control animals (P<0.05; Table 2). The remaining genes, Actn, GAPDH, Hg_Act1 and C5501, (that were found to be down-regulated in the microarray) were either significantly down-regulated in LPS-treated animals (Actn and C5501), or at least showed a tendency toward down-regulation (GAPDH and Hg_Act1) when analyzed by PCR. Thus, the results were consistent with those of the array presented on Table 1.

**Table 2:** Summary of array validations compared with the corresponding array result. Acsn #: NCBI's accession number of the corresponding EST.

EST ID	Acsn #	Homology	Array	p-value	RT-PCR	t-test
C5087-1	-	Myp	Up	0.00684	Up	0.0133
P7DP02C08	ES726499	Ahcy	Up	0.03773	Up	0.0209
PNLP17D06	ES729344	Frep	Up	0.03093	Up	0.038
P3DP22F02	ES725371	Actn	Down	0.04805	Down	0.0019
C4705-2	FJ455438	Hg_Act1	Down	0.0451	Down	0.19
P7DP32B09	ES727907	GAPDH	Down	0.0432	Down	0.25
PNLP09D04	ES728794	Unknown	Up	0.00552	Up	0.0302
P7AP03G08	ES725972	Unknown	Up	0.00684	Up	0.0123
C4874	-	Unknown	Up	0.00684	Up	0.0007
C5242	-	Unknown	Up	0.00684	Up	0.0051
C5501	-	Unknown	Down	0.02343	Down	0.0181

**Figure 5:**
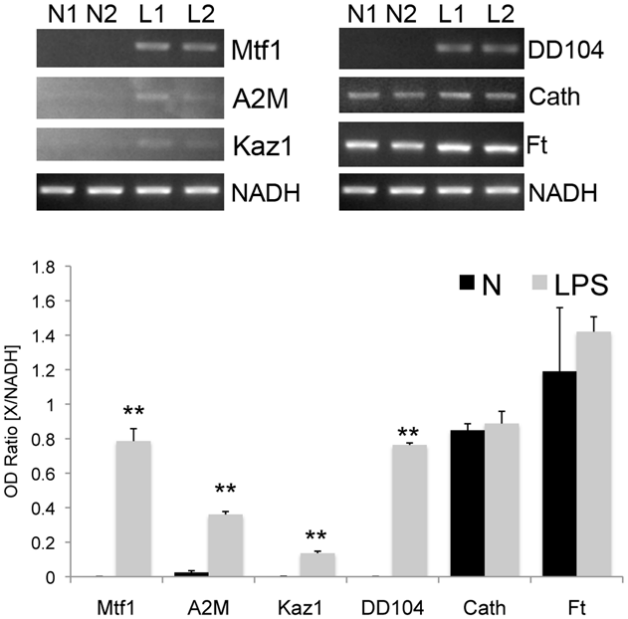
Semiquantitative relative RT-PCR of 6 immune associated genes. For each gene a representative gel image is shown, each lane represents RNA from a single individual. Graph bars indicate the averaged OD ratios between each gene and NADH for three different experiments (with three different animals). Lines represent standard deviation. Asterisks represent t-test significance (**P<0.01). Mtf1: Melanotransferrin 1 (accession number GQ243222); A2M: alpha-2-macroglobulin-like; Kaz1: Kazal-type serine proteinase inhibitor 1; Cath: Cathepsin; Ft: Ferritin (acc. No. EU010238).

We also explored the expression of genes (Mtf-1, DD104, Kaz1, A2M, Cath and Ft) that had been previously associated with the immune system [24] but that appeared to show no differential expression in the microarray. Interestingly, upon direct observation of the microarray data, we found that four of these genes (Mtf-1, DD104, Kaz1, A2M) had intensities over 2 times greater in LPS when compared to controls, but that the overall level of the intensities was rather low and not sufficient to consider the difference between control and LPS-injected to be significant. When the expression of these genes was validated with RT-PCR, we found that the same four, whose expression in the microarray appeared to be increased with LPS (Mtf-1, DD104, Kaz1 and A2M), showed significant up-regulation after LPS injection (Figure 5). Similarly, the two other genes that showed no differences in intensities between controls and LPS-injected animals in the microarray (Cath and Ft) also showed no difference in their expression levels when tested using RT-PCR (Figure 5).

## Discussion

We have induced the activation of the immune system in holothurians by a systemic injection with LPS and focused on the response of the intestinal tissues in terms of gene expression. Gene expression was assessed using microarray technology showing that at least 50 genes are differentially expressed between intestines of LPS-injected and vehicle-injected controls. Lipopolysaccharide (LPS) is the most used immune-activating substance, whose effects are not only seen in lymphocytes (and related immune cells) but in other tissues as well. A classical example is the induction of fever in mammals, in which a systemic (intra-peritoneal or intravenous) injection of LPS provokes central nervous system responses leading to an increase in body temperature as well as other behavioral changes [34]. Thus, LPS has been widely used as a tool to study immune-responsive genes in vertebrates and invertebrates, and even in plants [35], [36]. Additional evidence from invertebrate models shows the effect of LPS on the expression levels of immune genes in different tissues. For example, LPS induces the expression of a tachylectin-related protein in the gut of amphioxus [37], an anti-LPS factor in the lymphoid organ of prawn [38], TNFα homologues in the pharynx of Ciona [39] and in the gills of a mollusk [40]. In addition, a previous publication from our group showed the up-regulation of an holothurian homologue of the Serum amyloid A (SAA) gene in the intestine after an LPS injection [41].

### The microarray

The arrays performed as expected, imparting a high degree of confidence in the obtained data and assuring that further statistical analyses were free of technical variability. Observed variations might then be due to biological changes/variation or small (undefined) differences between the treatments.

However, there are limits to the microarray detection and this is obvious when we observed the expression profiles of immune genes with lower signal intensities. The apparent differential expression between normal and LPS injected animals was not significant using in the microarray analysis. However, other more sensitive techniques, such as RT-PCR, or Northern blots, demonstrated differential expression. This also appears to be the case for holothurian Serum Amyloid A gene (SAA). This gene had been previously shown by our group to be induced by LPS in the intestine using Northern blots [41]. Although the microarray statistical analysis did not show a significant change, when we compared the SAA probe intensities between LPS and control intestines, the LPS-treated animals showed an average intensity 3 times greater than the controls. Nonetheless, the intensity of these spots was not sufficiently high to be statistically significant. This is concordant with our northern blot data [41] showing that expression of this gene in the LPS-treated intestine although higher than in controls is still quite low. Therefore, the microarray results might be biased toward those genes that show high intensities (high expression) and therefore the real number of differentially expressed sequences might be higher than those determined by the microarray.

Finally, the microarray validations using semiquantitative RT-PCR corroborated the performance of the microarray providing strong support to the microarray results. From 11 chosen genes for validation, 9 showed the same expression profiles revealed by the array with statistical significance. The remaining 2 genes, although not significant probably due to the small number of samples, followed a tendency that was similar to the results from the array.

### Differentially expressed genes

The seven differentially-expressed genes that can be identified by their homology provide an initial glance into the events and molecules that might be associated with an intestinal response to bacterial attack. Thus, we can use their expression profiles to speculate about the possible roles these genes are playing in the intestinal immune response. Following is a discussion of what is known about the genes and their relationship with immune responses. The genes have been grouped in accordance to their main known function.

### Metal ion metabolism/transport genes

The upregulation of holothurian Myp after LPS injection indicate that this gene may have an immune role. The sea urchin MYP was first identified as a vitellogenin, due to its presence in the egg and the yolk platelet [42], [43], [44]. This protein does not fit the profile of a common vitellogenin however, mainly because it is not restricted to females, is not developmentally regulated [43], [45], and it accounts for 50% of the total protein content in the coelomic fluid [46]. Its presence in the coelomic fluid suggests a possible immune function. Moreover, the sea urchin MYP (and also the holothurian) possesses transferrin-like domains, which can bind iron and other metal ions; in vitro assays have demonstrated that the sea urchin MYP binds iron [46] and zinc [47]. Since iron is an essential element for bacterial metabolism, the binding capacity of MYP gives it bacteriostatic properties. The main source of the sea urchin MYP is the gut [46], [48], in accordance with the expression profile found in *H. glaberrima*. The up-regulation of Myp mRNA after LPS challenge could indicate an increase in MYP delivery to coelomocytes and coelomic fluid, to sequester iron and help fighting against an invading pathogen.

### Cellular metabolism genes

Three genes for metabolic enzymes were found to be differentially expressed according to the array, Ahcy, Gnmt and Gapdh. The first two are key enzymes that regulate cellular transmethylation reactions due to the inhibition of other methyltransferases by their substrates and product [49], [50], [51]. The inhibition of transmethylation reactions mostly affects the efficiency of gene expression, from the methylation of the promoters, cap formation, the stability and export of mRNA, to the initiation of translation. The induction of Ahcy and Gnmt mRNAs by LPS in intestinal tissues could indicate an increase in transmethylation metabolism. This increment could indirectly reflect an increase in gene expression, methylation, and de-methylation of DNA and cell proliferation. Ahcy also has been implicated in chemotaxis [52] and immune function [53], two roles that also fit the profile of this holothurian homologue. In *Dyctiostelium* and human neutrophils, this protein is asymmetrically distributed towards the front of chemotacting cells, and its inhibition impairs chemotaxis [52]. Therefore, up-regulation of Ahcy may be indicative of increasing chemotaxis in intestinal cells of the holothurian. However, experiments to determine the spatial expression and localization of this gene are needed to corroborate this hypothesis. On the other hand, experiments in rat and mouse, link Ahcy to immune activation of T-cells and macrophages, and its inhibition reduces inflammation and impairs t-cell proliferation. This is mostly due to the high dependence of T-cells on methylation reactions for proliferation and maturation and the interaction of Ahcy with second messengers in macrophages that regulate their activation and phagocytic activity [53]. These processes can also occur in holothurians, suggesting an immune role for Ahcy in activation of phagocytic coelomocytes or perhaps proliferation of other cell types in the intestine.

In contrast with Ahcy and Gnmt, the other metabolic enzyme gene, Gapdh appeared down-regulated on the microarray. GAPDH is an enzyme that catalyzes the sixth step of glycolysis for energy production. In addition to this metabolic function, GAPDH has also been implicated in several non-metabolic processes, including transcription activation [54], initiation of apoptosis [55], and ER to Golgi vesicle shuttling [56]. GAPDH also acts as a reversible metabolic switch during oxidative stress; its inactivation temporarily re-routes the metabolic flux towards the production of more NADPH, as is needed by some antioxidant systems [57]. Since LPS causes oxidative stress [58], [59], the holothurian intestine could respond to a bacterial challenge by down-regulating GAPDH expression. A similar response was also found in Drosophila hemocytes, where GAPDH levels were found to be reduced across different LPS doses [60]. However, more evidence is still needed to confirm the expression patter of holothurian GAPDH, first, at the protein level to effectively prove a downregulation of GAPDH, and second, testing reactive oxygen species (ROS) production to verify oxidative stress in the intestine after LPS. Although the RT-PCR validation of this expression profile was not statistically significant, a tendency towards down-regulation is evident.

### Cytoskeletal genes

Two genes in this category were found to be down-regulated in LPS-treated intestines: alpha-actinin (Actn) and an isoform of actin identified as Actin 1 in *H. glaberrima* (Hg_Act1). Actin proteins are highly conserved cytoskeletal proteins found all over the eukaryote domain. They are important cytoskeletal proteins involved in a broad range of cellular processes, from movement to cell division [61], [62], [63]. Alpha-actinin is an actin-binding protein that cross-links actin filaments [64]. It is involved in stabilization of cell junctions and as a link between the cytoskeleton and transmembrane proteins [65]. Chicken alpha-actinin has also been found to be down-regulated in intestinal lymphocytes after challenge with two protozoa [66], suggesting that a similar mechanism may be occurring in the sea cucumber. Several actin-binding proteins were downregulated in Drosophila hemocytes activated with LPS [67], indicating that immune cells may responding to LPS by remodeling the cytoskeleton. The expression profile of these two cytoskeletal proteins in the holothurian suggests two hypotheses: First, that down-regulation of this actin isoform may be indicative of changes in cytoskeleton dynamics, probably the disassembling of the actin network; and second, down-regulation of alpha-actinin may be related to changes in cell adhesion and motility. A study about cell de-adhesion dynamics showed that this process requires changes in stress fibers and focal adhesions, which includes the loss of certain structural proteins, being alpha-actinin one of them [68]. These changes may occur in the cells of the coelomic epithelium that are in direct contact with the coelomic fluid (where the LPS was injected) or in wandering coelomocytes residing in the intestinal tissues. These cytoskeletal changes could be linked to chemotaxis of local coelomocytes and/or detachment of adherens junctions of some intestinal cells. Another link can be made to the phagocyte increase after LPS challenge, where some of these cells might be detaching from the intestinal coelomic epithelium and migrating to the coelom.

### Recognition/defense genes

Previously, we reported 7 ESTs with fibrinogen-like domains that may be part of the immune repertoire of *H. glaberrima* [24]. One of these ESTs (PNLP17D06) was found to be up-regulated in the intestine after LPS injection. The fibrinogen-like domain is present in other proteins besides fibrinogen, i.e. ficolins, tenascins, angiopoietins, fibrinogen-related proteins (FREPs) to name a few. With the current sequence information for the holothurian fib-like proteins it was still not possible to determine the identity for each of the 7 ESTs. However, it is very likely that these proteins may have an immune role. The induction of PNLP17D06 in the intestine by LPS corroborates this. The fibrinogen-like domain has the ability to interact with other protein domains, and several proteins with this domain have recognition capabilities. Some of them take part in immune responses in several organisms. For example, ficolins have lectin-like properties through their fibrinogen-like domain, and they participate in innate immune reactions [69]. FREPs play an important role in the innate immune response of snails and mosquitoes against pathogens [70], [71], vertebrate FREPs also have immune functions, taking part in acute phase reactions and regulatory T-cell activity [72], [73]. Therefore this holothurian EST (PNLP17D06) bearing the fib-like domain, may represent an example of one of these molecules, exerting its intestinal function of recognizing pathogens.

### Comparative studies

Previous studies using LPS to activate the echinoderm immune system have been done to determine changes in gene expression in both sea urchin and sea cucumber coelomocytes. The results of these studies provide a list of differentially expressed genes that serve as a frame of reference for echinoderm immunology and that can be used to compare our results [20], [21], [24]. This comparison, summarized on Table 3, provides a glimpse that suggests both organ-specific responses and inter-species differences. In general, there is some correlation between the holothurian coelomocytes and intestinal immune gene expression, i.e. genes differentially expressed after LPS in holothurian coelomocytes also change in the intestine. On the other hand, genes with different expression profiles after LPS challenge in both tissues represent unique ways for each organ to respond to bacterial infection. For example, the intestine up-regulates Myp and FREP, while coelomocytes do not change the levels of those genes (unpublished results).

**Table 3:** Comparison of genes differentially expressed following and LPS challenge in the sea urchin *S. purpuratus* [20], [21] and the sea cucumber *H. glaberrima* [24].

Genes	Sea cucumber		Sea urchin
	Intestine	Coelomocytes	Coelomocytes
DD104	Up(†)	Up	Up
C-type lectin	n.c.	n.c.	Up
Cathepsin	n.c.	n.c.	Up
Thymosin	n.c.	n.c.	Up
Actin	Down	n.c.(*)	Up
Kazal-1	Up(†)	Up	-
Mtf1	Up(†)	Up	-
SAA	Up(†)	Up	-
A2M	Up(†)	Up	-
Myp	Up	n.c. (*)	-
FREP	Up	n.c.(*)	-

n.c: no change;

† no change on the microarray, upregulation shown by RT-PCR;

* unpublished data.

Moreover, there is little correlation between the LPS-activated coelomocytes of the two species, with only one out of five genes upregulated following LPS. This gene, DD104 which remains fairly uncharacterized, is readily induced by a bacterial challenge and injury in sea urchin coelomocytes [74] and by LPS in holothurian coelomocytes [24] and intestine. The differences in coelomocyte expression between species might be due to the immunological status of the animals. In the sea urchin experiments, animals were kept in the lab for at least six months until they reached an immunoquiescent status. In our experiments, animals were obtained directly from the field, kept in the lab for a week and then immune activated. Therefore, it is possible that our animals are partially immune activated in nature and thus no differences are observed for some of the genes that were found to be activated in the immunoquiescent sea urchins when exposed to LPS. Another possibility is that the seawater injection used in the controls provoked an immune response per se. This has been documented in sea urchin, where the injury of the injection along with ionic changes due to the sea water entry into the coelom, can mimic the effects of a bacterial infection [75]. Thus, if this also occurs in holothurians, the seawater injections in controls may have reduced the amount of differentially expressed genes found by our microarray.

### Unknown genes

So far, we have analyzed the genes that had some similarity to other genes in the databases, but these genes only represent 14% of all differentially expressed genes in the microarray (7 out of 50). The remaining 86% (43 ESTs) correspond to sequences with no evident similarities in protein, EST, or RNA databases. Twenty-nine (29) of these sequences did not have a recognizable open reading frame (ORF), these may represent untranslated regions (UTRs) for long transcripts whose ORFs were not incorporated when the library was done, or may also be regulatory non-coding RNAs (ncRNAs) which may be regulating the expression of other genes [76], [77]. Eight of the 29 ESTs had a polyadenylation signal confirming that they are 3′UTRs. For these ESTs, 5′RACE experiments are needed to get into the ORF and determine the identity of the transcripts. The remaining 21 ESTs need further sequence characterization to determine if they are part of a longer UTR or if they are ncRNAs.

On the other hand, the fourteen (14) remaining ESTs did show a recognizable ORF. The conceptual translation of these ORFs did not result in sequences with domains or motifs. Thus, these sequences may correspond to unconserved protein regions. More sequence information is needed to either find a domain or sequence that helps identify them or characterize them as novel genes. These unknown sequences represent an excellent opportunity to find immune-related genes. In fact the 185/333 family of genes in the sea urchin were initially found as an unknown EST (encoding for an uncharacterized protein) after sequencing clones from a cDNA library of LPS-activated coelomocytes [21]. Further analysis revealed a complex and dynamic family of transcripts with unmistakable immune functions that appears to be echinoderm (and maybe echinoid) specific [78], [79]. Therefore, efforts are in progress to fully characterize these unknown sequences and to determine their identity and relationship to the immune response of holothurians.

Finally, we cannot possibly suggest that our results comprise all the intestinal genes associated with the immune response. First, because we only used one immune activator (LPS) and one time point for comparison. Second, because we are comparing animals collected from their natural habitats to the same group of animals following an LPS injection. Although we have previously shown that these animals respond to LPS by increasing their phagocytosis up to 25% [23] (thus they are still capable of increasing their immune activation) they might be partially immune activated in nature. Third, because the probes on the microarray chip were limited to around 7000 ESTs obtained from our cDNA libraries, and other immune genes might be present in the holothurian genome. Finally, because as our results have shown, there are some technical limits to the microarray, in particular concerning those sequences that show low expression intensities. Nevertheless, our results provide a first approach and some interesting discoveries to the issue of immune activation in non-immune organs and to the echinoderm immune system in general.

### Summary

We have shown that a systemic injection of LPS in the sea cucumber *H. glaberrima* provokes changes in the expression of several intestinal transcripts. This expression profile seems to be organ-specific when compared to expression profiles of coelomocytes activated with LPS in both the holothurian or in the sea urchin.

Genes that were up-regulated by LPS in the holothurian intestine include: Myp, a gene related to iron transport, which may be involved in intestinal immune responses by acting as an iron-sequestering bacteriostatic protein. Also, two genes for proteins involved in transmethylation metabolism, Ahcy and Gnmt. This induction may reflect an increase in several cellular events closely tied to transmethylation metabolism, such as gene transcription, cell proliferation or even the activation of phagocytes. LPS also induced the expression of a fibrinogen-related protein gene, which acting as a pattern recognition protein may be synthesized to recognize pathogens.

On the other hand, LPS injection caused downregulation of the mRNA levels for GAPDH, perhaps as a response to oxidative stress induced by LPS. In addition, two cytoskeletal proteins were also downregulated in the LPS-treated intestine, Actin and Alpha-actinin, probably reflecting changes in cytoskeletal dynamics. These cytoskeletal changes may be directed towards cell detachment, and increased mobility perhaps by intestinal cells migrating towards the coelomic cavity, where the challenge was presented.

The majority of differentially expressed genes were unknown genes with no homologues in the databases. These genes may represent novel genes or regulatory ncRNAs highly responsive to LPS, either by their induction or repression.

In conclusion, a wide variety of mRNAs were expressed by the intestine in response to the immune challenge presented by LPS. Our results open a new door in terms of new unidentified molecules, specific species and organ responses and in particular on the immune interactions of the intestine and its response to an LPS challenge.

## Supporting Information

Table S1 Primers used for semiquantitative relative RT-PCR
(0.01 MB PDF)

Table S2 Differentially expressed probes with their respective EST match. Shaded in grey are the p-values <0.01. Acsn #: NCBI's accession number for the corresponding EST.
(0.08 MB PDF)

Figure S1 Agilent's SpikeIns performance in the sea cucumber microarrays. A. Comparation of expected signals for 10 spikeIns versus the observed signals in the array. Letters at the end of each row represent the expected color of the spot on the array (BG: bright green, DG: dark green, Y: yellow, O: orange, R: red). SD: standard deviation. B. Linear regression of the expected vs observed logRatios of all the spikeIns in the array. C. Image of 5 spikeIns and one negative control (RC8) on the array.
(9.77 MB TIF)

Figure S2 MA Plots of raw unnormalized data (superior box) and normalized data of the microarray (inferior box).
(7.74 MB TIF)

Figure S3 Area of the N vs. LPS microarray showing the color change in dye swaps experiments. Arrowheads show some differentially expressed genes. Arrows show the Agilent internal controls for color and intensity. Lower corner/white line provides orientation.
(2.05 MB TIF)
