# Supplementary material for: LPS-Induced Genes in Intestinal Tissue of the Sea Cucumber Holothuria glaberrima
Source: PLoS One. 2009 Jul 8;4(7):e6178. doi: 10.1371/journal.pone.0006178 (PMC2702171; doi:10.1371/journal.pone.0006178)
Supplement: Table S1 — Primers used for semiquantitative relative RT-PCR (0.01 MB PDF) [file pone.0006178.s001.pdf]

Table S1. Primers used for semiquantitative relative RT-PCR

Gene	Forward and Reverse primer	Tm	Product size
NADH	CGGCTACTTCTGCGTTCTTC	60	241
	ATAGGCGCTGTCTCACTGGT	59	
Ahcy	TGGGGATGTTGGTAAGGGTA	60	432
	TGGTTGGTGAAGGAGTTTGA	59	
Myp	ATGACTGGTGGAAGGAGAGG	59	436
	TTGGTATTGAGGGTGTGGTT	57	
Hg_Act1	ATCGTGTTGCATTCGTGTTG	58	200
	TCCTTCAGGTGGTCAGTCCT	62	
GAPDH	GAAAGGTGCCAAGTATGAGGA	62	472
	GCAAAATCACAAACAGGGG	56	
Actn-2	TTGGGAGAGGCAACAGAAG	58	327
	TGAATGGCAAAACGGAGG	54	
Frep	AATGTCTTCCGTATCACCG	56	294
	AAAACCGTCCATCCACCT	54	
C4874	AAATGGCCCTCCCTACCTC	60	482
	TATGCTTCCTCACCCAATCC	60	
C5501	AAACACAGGAGGTTGGGGA	58	448
	ACGCTGGTGGCTTTCTTG	56	
C5242	GCAGTAAGAGACAGTAACGAAACA	68	294
	AGAAATAGAAAGACTCCCACCAC	66	
PNLP9D4	TTTAAGCGAAAATGTCCATGC	58	459
	TGTGGAAAGAGAAAAACTGCAA	60	
P7AP3G8	TGGTCACAGTATCCGGTTTTC	62	397
	GCTGCGCTAGTTTCGCTATC	62	
