# Supplementary material for: LPS-Induced Genes in Intestinal Tissue of the Sea Cucumber Holothuria glaberrima
Source: PLoS One. 2009 Jul 8;4(7):e6178. doi: 10.1371/journal.pone.0006178 (PMC2702171; doi:10.1371/journal.pone.0006178)
Supplement: Table S2 — Differentially expressed probes with their respective EST match. Shaded in grey are the p-values <0.01. Acsn #: NCBI's accession number for the corresponding EST. (0.08 MB PDF) [file pone.0006178.s002.pdf]

Table S2. Differentially expressed probes with their respective EST match. Shaded in grey are the p-values <0.01. Acsn #: NCBI's accession number for the corresponding EST

Upregulated genes

Genes.ProbeName	EST (Target)	Acsn #	p.value
CUST_8666_PI378611823	PNLP09D04040_5229	ES728794	0.00552
CUST_896_PI378625531	PNLP09D04040_5229	ES728794	0.00552
CUST_934_PI378611823	P3DP29C02026_9750	ES725707	0.00684
CUST_7700_PI378611823	P7AP03G08080_1983	ES725972	0.00684
CUST_7781_PI378611823	P7AP03G08080_1983	ES725972	0.00684
CUST_10533_PI378611823	P7DP08F05065_4117	ES726825	0.00684
CUST_9074_PI378611823	P7DP08F05065_4117	ES726825	0.00684
CUST_855_PI378611823	P7DP22G06078_7207	ES727421	0.00684
CUST_746_PI378611823	P7DP31B01013_10485	ES727831	0.00684
CUST_1585_PI378625531	PNLP03D08044_4729	ES728487	0.00684
CUST_561_PI378611823	PNLP03D08044_4729	ES728487	0.00684
CUST_11961_PI378611823	PNLP09D04040_5229	ES728794	0.00684
CUST_5512_PI378611823	PNLP20D05041_7929	ES729555	0.00684
CUST_9021_PI378611823	PNLP25B05017_9033	ES729873	0.00684
CUST_881_PI378611823	PNLP03D08044_4729	ES728487	0.00703
CUST_3239_PI378611823	P7DP22G06078_7207	ES727421	0.00726
CUST_6807_PI378611823	PNLP03D08044_4729	ES728487	0.00726
CUST_12028_PI378611823	P3DP19C11035_442	ES725198	0.00762
CUST_2032_PI378625531	P7AP03G08080_1983	ES725972	0.00933
CUST_1954_PI378625531	P3DP19C11035_442	ES725198	0.01062
CUST_8643_PI378611823	P7DP22G06078_7207	ES727421	0.01062
CUST_4613_PI378611823	PNLP06E09057_5075	ES728660	0.01062
CUST_716_PI378611823	PNLP13E06054_10273	ES729033	0.01062
CUST_1616_PI378625531	PNLP13E06054_10273	ES729033	0.01071
CUST_2010_PI378611823	P7DP14H09093_10057	ES727089	0.0124
CUST_8724_PI378611823	P3DP19C11035_442	ES725198	0.01553
CUST_9140_PI378611823	P7DP28H09093_8325	ES727687	0.01675
CUST_6035_PI378611823	P7DP31B01013_10485	ES727831	0.01676
CUST_2386_PI378625531	PNLP06E09057_5075	ES728660	0.01759
CUST_7119_PI378611823	PNLP13E06054_10273	ES729033	0.01984
CUST_9115_PI378611823	P3DP29C02026_9750	ES725707	0.0199
CUST_7655_PI378611823	P7DP05H09093_3819	ES726711	0.0199
CUST_7306_PI378611823	P7DP05H09093_3889	ES726711	0.0199
CUST_414_PI378611823	PNLP26B05017_9165	ES729939	0.0199
CUST_4803_PI378611823	PNLP02G11083_4641	ES728443	0.02028
CUST_3640_PI378611823	PNLP20G12084_7987	ES729584	0.0209
CUST_6091_PI378611823	PNLP19B04016_7767	ES729474	0.02106
CUST_12993_PI378611823	PNLP02G11083_4641	ES728443	0.02171
CUST_11172_PI378611823	PNLP20D05041_7929	ES729555	0.02649

CUST_8964_PI378611823	PNLP06E09057_5075	ES728660	0.02659
CUST_1980_PI378625531	P7DP28H09093_8325	ES727687	0.02669
CUST_11414_PI378611823	PNLP21H10094_8597	ES729655	0.02669
CUST_1015_PI378611823	PNLP20G12084_7987	ES729584	0.02717
CUST_3422_PI378611823	P7DP21E08056_7047	ES727341	0.02932
CUST_6743_PI378611823	PNLP14B02014_5631	ES729080	0.02932
CUST_10849_PI378611823	P7DP04E06054_3671	ES726637	0.0309
CUST_4193_PI378611823	P7DP14H09093_10057	ES727089	0.03093
CUST_415_PI378611823	P7DP21E08056_7047	ES727341	0.03093
CUST_10018_PI378611823	PNLP06E09057_5075	ES728660	0.03093
CUST_3950_PI378611823	PNLP17D06042_6167	ES729344	0.03093
CUST_5809_PI378611823	PNLP26F10070_9253	ES729983	0.03093
CUST_8194_PI378611823	PNLP12E07055_5605	ES728982	0.0313
CUST_12133_PI378611823	P7AP01A08008_1641	ES725801	0.03335
CUST_1912_PI378625531	PNLP17D06042_6167	ES729344	0.03553
CUST_341_PI378625531	PNLP12E07055_5605	ES728982	0.03687
CUST_7813_PI378611823	P7DP02C08032_3395	ES726499	0.03773
CUST_5489_PI378611823	P7AP07G04076_2449	ES726205	0.03899
CUST_3977_PI378611823	P7AP02B05017_1779	ES725870	0.0451
CUST_2253_PI378625531	P7DP02C08032_3395	ES726499	0.04564
CUST_1658_PI378625531	PNLP07A02002_10065	ES728683	0.04564
CUST_11810_PI378611823	PNLP07A02002_10065	ES728683	0.04658
CUST_2546_PI378625531	P7DP21E08056_7047	ES727341	0.04743
CUST_587_PI378611823	P3DP09E03051_1520	ES724847	0.04761
CUST_7738_PI378611823	P7DP02C08032_3395	ES726499	0.0477
CUST_8487_PI378611823	P7DP28H09093_8325	ES727687	0.0477
CUST_6134_PI378611823	P7DP21E08056_7047	ES727341	0.04867
CUST_2121_PI378611823	PNLP26A01001_9137	ES729925	0.04963

Downregulated genes

Genes.ProbeName	EST (Target)	Acsn #	p.value
CUST_7374_PI378611823	P7DP34E01049_11003	ES728090	0.0124
CUST_2858_PI378611823	P7AP07B02014_2393	ES726177	0.01613
CUST_181_PI378611823	P7DP02G09081_3459	ES726531	0.01613
CUST_11511_PI378611823	PNLP17C12036_6155	ES729338	0.01613
CUST_2445_PI378625531	PNLP17C12036_6155	ES729338	0.01613
CUST_1892_PI378625531	P7DP34E01049_11003	ES728090	0.0209
CUST_3012_PI378611823	P3DP18G01073_378	ES725166	0.02343
CUST_8007_PI378611823	P7DP34E01049_11003	ES728090	0.02343
CUST_2283_PI378625531	P3DP18G01073_378	ES725166	0.0245
CUST_30_PI378625531	P3DP09F12072_1536	ES724855	0.02668
CUST_4942_PI378611823	P7DP08F07067_4121	ES726827	0.02668
CUST_1034_PI378611823	P7DP42F11071_11397	ES728287	0.02668
CUST_12166_PI378611823	P3DP09F12072_1536	ES724855	0.02669

CUST_10072_PI378611823	P3DP26D06042_9424	ES725544	0.02669
CUST_10085_PI378611823	P7DP08F07067_4121	ES726827	0.02669
CUST_12889_PI378611823	PNLP10F03063_5393	ES728876	0.02669
CUST_347_PI378625531	PNLP26B02014_9159	ES729936	0.02669
CUST_8572_PI378611823	P3DP18D04040_340	ES725147	0.02932
CUST_6001_PI378611823	P7DP42F11071_11397	ES728287	0.0309
CUST_1939_PI378611823	PNLP26B02014_9159	ES729936	0.0309
CUST_3011_PI378611823	P3DP18G01073_378	ES725166	0.03107
CUST_10319_PI378611823	P7DP20E01049_6925	ES727280	0.03335
CUST_8008_PI378611823	P7DP34E01049_11003	ES728090	0.03335
CUST_2377_PI378625531	P7DP42F11071_11397	ES728287	0.03335
CUST_2989_PI378611823	P3DP01B04016_934	ES724555	0.03483
CUST_2636_PI378625531	PNLP01B07019_4399	ES728322	0.035
CUST_5602_PI378611823	P7DP02G09081_3459	ES726531	0.03571
CUST_456_PI378611823	PNLP17C12036_6155	ES729338	0.03632
CUST_2768_PI378611823	P7DP34E01049_11003	ES728090	0.03687
CUST_12167_PI378611823	P3DP09F12072_1536	ES724855	0.03773
CUST_2584_PI378611823	P3DP14F03063_84	ES724974	0.03773
CUST_7563_PI378611823	P7DP32C11035_10661	ES727919	0.03781
CUST_4670_PI378611823	P7DP25G11083_7535	ES727585	0.03786
CUST_8573_PI378611823	P3DP18D04040_340	ES725147	0.03847
CUST_8844_PI378611823	P3DP15A05005_100	ES724982	0.03899
CUST_7752_PI378611823	P7DP01C01025_3269	ES726436	0.03899
CUST_13131_PI378611823	P7DP34B09021_10951	ES728064	0.03899
CUST_8611_PI378611823	PNLP20G10082_7985	ES729583	0.03899
CUST_12985_PI378611823	PNLP13E12060_10283	ES729038	0.03965
CUST_144_PI378625531	P7DP01C01025_3269	ES726436	0.04251
CUST_3292_PI378611823	PNLP12D09045_5589	ES728974	0.04278
CUST_10559_PI378611823	P7DP30C11035_10401	ES727789	0.04318
CUST_7053_PI378611823	P7DP32B09021_10637	ES727907	0.04318
CUST_11773_PI378611823	PNLP10F05065_5397	ES728878	0.04318
CUST_9438_PI378611823	P3DP27E03051_9532	ES725598	0.0451
CUST_597_PI378611823	P7AP11C04028_2913	ES880959	0.0451
CUST_2619_PI378625531	P7DP08F07067_4121	ES726827	0.0451
CUST_2630_PI378611823	P7AP03D09045_1935	ES725948	0.04523
CUST_2189_PI378611823	P3DP26D06042_9424	ES725544	0.04564
CUST_546_PI378625531	P7AP07G08080_2457	ES726209	0.04564
CUST_4468_PI378611823	P7AP09A02002_2587	ES726274	0.04564
CUST_4247_PI378611823	P7DP27E11059_7707	ES727671	0.04564
CUST_1016_PI378611823	PNLP11E04052_5491	ES728925	0.04564
CUST_12664_PI378611823	P3DP15F10070_168	ES725016	0.04658
CUST_8472_PI378611823	P7DP08H09093_4161	ES726847	0.04658
CUST_2500_PI378625531	P7DP25G11083_7535	ES727585	0.04676
CUST_6127_PI378611823	P3DP14F03063_84	ES724974	0.0477
CUST_536_PI378611823	P3DP22F02062_788	ES725371	0.04805
CUST_4589_PI378611823	P7DP29A04004_8337	ES727691	0.04805
CUST_9873_PI378611823	P7DP32B09021_10637	ES727907	0.04805
CUST_9343_PI378611823	PNLP01G05077_4473	ES728359	0.04805

CUST_12183_PI378611823	P7DP08H09093_4161	ES726847	0.04905
CUST_773_PI378625531	PNLP17B07019_6129	ES729325	0.04905
CUST_10448_PI378611823	P3DP29G02074_9805	ES725735	0.04963
CUST_655_PI378611823	P7DP10D05041_4299	ES726915	0.04963
CUST_1146_PI378611823	P7DP32C11035_10661	ES727919	0.04963
CUST_8579_PI378611823	PNLP17B07019_6129	ES729325	0.04963
CUST_4604_PI378611823	P7AP05D03039_2161	ES726128	0.04972
CUST_8612_PI378611823	PNLP20G10082_7985	ES729583	0.04972
