# Supplementary figures and images for: LPS-Induced Genes in Intestinal Tissue of the Sea Cucumber Holothuria glaberrima
Source: PLoS One. 2009 Jul 8;4(7):e6178. doi: 10.1371/journal.pone.0006178 (PMC2702171; doi:10.1371/journal.pone.0006178)

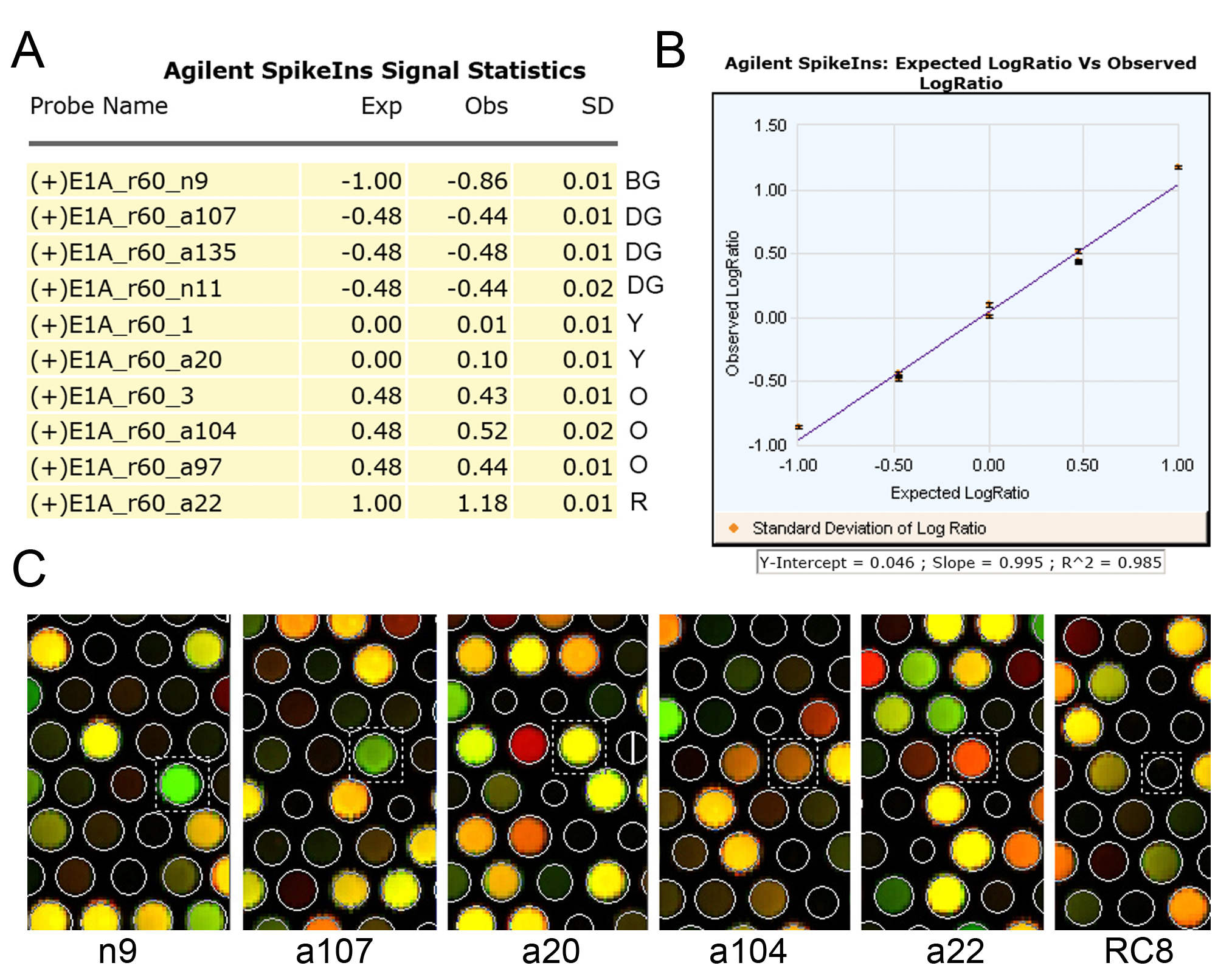

Supplement: Figure S1 — Agilent's SpikeIns performance in the sea cucumber microarrays. A. Comparation of expected signals for 10 spikeIns versus the observed signals in the array. Letters at the end of each row represent the expected color of the spot on the array (BG: bright green, DG: dark green, Y: yellow, O: orange, R: red). SD: standard deviation. B. Linear regression of the expected vs observed logRatios of all the spikeIns in the array. C. Image of 5 spikeIns and one negative control (RC8) on the array. (9.77 MB TIF) [file pone.0006178.s003.tif]

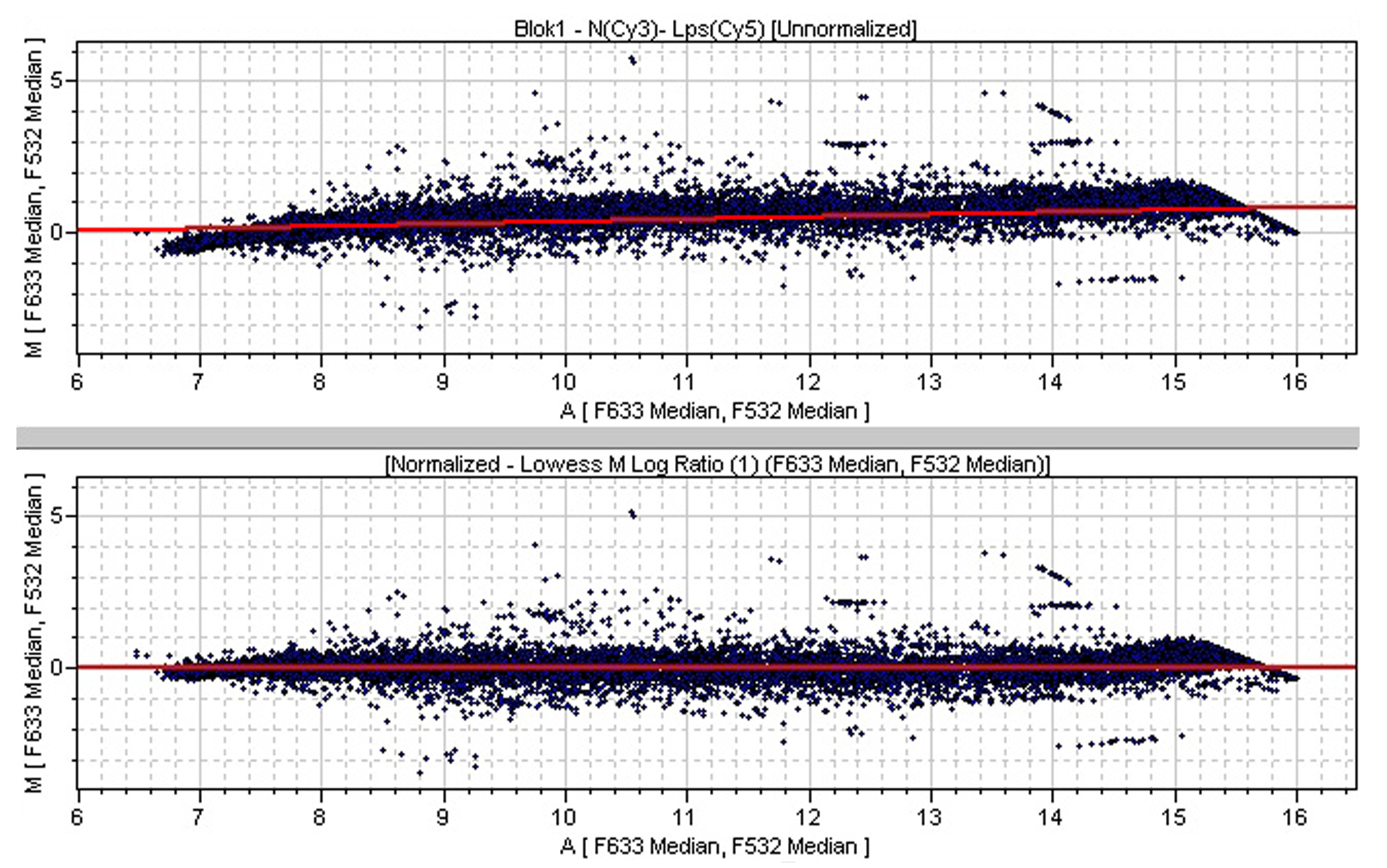

Supplement: Figure S2 — MA Plots of raw unnormalized data (superior box) and normalized data of the microarray (inferior box). (7.74 MB TIF) [file pone.0006178.s004.tif]

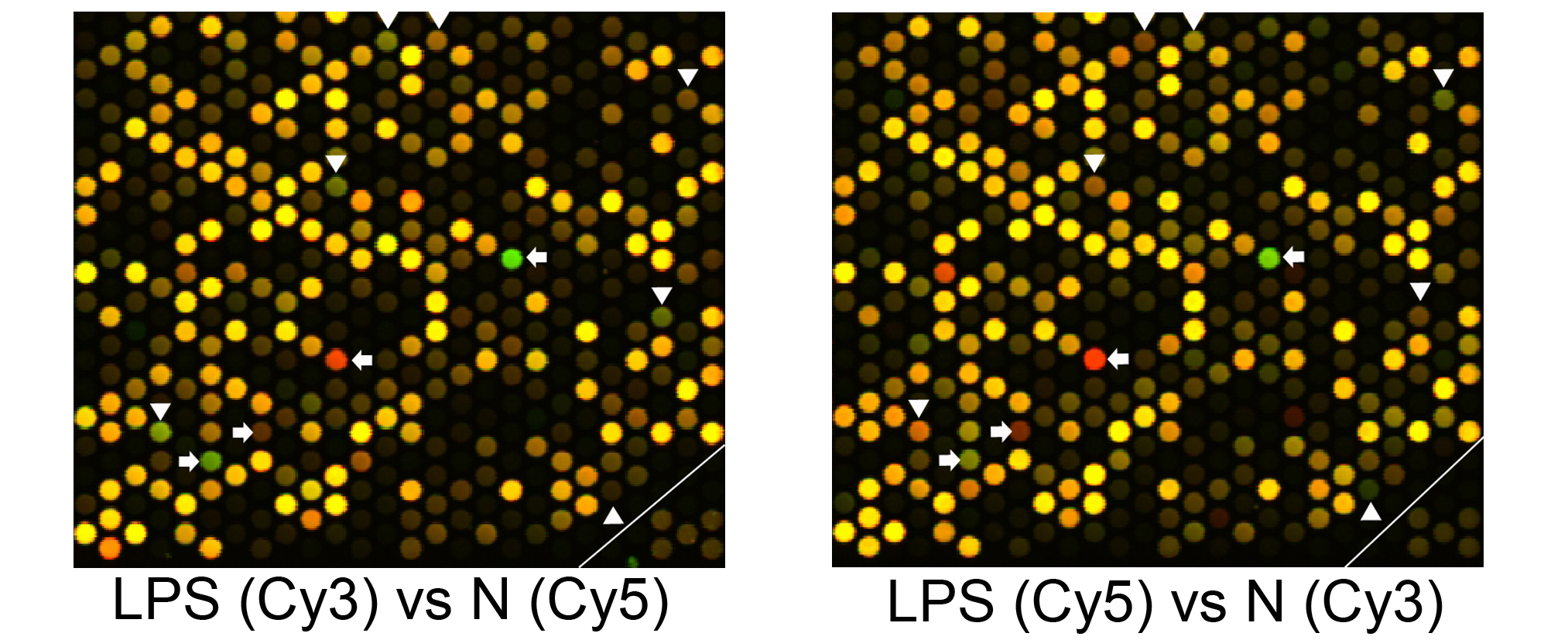

Supplement: Figure S3 — Area of the N vs. LPS microarray showing the color change in dye swaps experiments. Arrowheads show some differentially expressed genes. Arrows show the Agilent internal controls for color and intensity. Lower corner/white line provides orientation. (2.05 MB TIF) [file pone.0006178.s005.tif]
